# Anti-microbial activity of *Ocimum sanctum L.* gel against black pigmented microbes

**DOI:** 10.6026/973206300200277

**Published:** 2024-03-31

**Authors:** Jaiganesh Ramamurthy, Burra Anand Deepika

**Affiliations:** 1 Department of Periodontics, Saveetha Dental College and Hospitals, Saveetha Institute of Medical And Technical Sciences (SIMATS), Saveetha University, Chennai, Tamil Nadu, India

**Keywords:** Periodontitis, bacteria, micro-organisms, oral anaerobes, black pigmented, bacteroides, *Ocimum sanctum.L* (Tulsi)

## Abstract

Black pigmented gram negative anaerobes are associated with periodontal disease and tooth loss. Therefore, it is of interest to evaluate the antimicrobial activity of *Ocimum Sanctum.L* (Tulsi) gel against black pigmented anaerobes. Plaque samples were collected from the subject and kept in anaerobic broth for 4 hours of incubation at 37°C. 50µL concentration of Tulsi gel was added and kept in gas pack system for 3-5 days. Zone of inhibition was measured. *Ocimum sanctum L.* (Tulsi) exhibits strong antibacterial activity against Black Pigmented bacteroides at 1% and 2%.Tulasi gel was effective at higher concentrations, indicating the possibility of using it as an adjunct to standard periodontal treatment.

## Background:

Periodontitis is host-mediated and microbial associated inflammation that leads to loss of periodontal attachment. The disease's pathophysiology has been characterized in terms of its major molecular pathways, which ultimately result in the activation of host-derived proteinases. [1] It also leads to loss of marginal periodontal ligament fibers, apical migration of the junction epithelium, and apical spread of the bacterial biofilm along the root surface. [2] It also occurs as inflammation, the spread of inflammation from epithelium to connective tissue takes place laterally and apically resulting in the destruction of collagen fibers. When collagen fibers are destroyed, gingivitis progresses to periodontitis, which is clinically characterized as "attachment loss". Gradually due to the activation of osteoclast cells, bone resorption is initiated leading to gradual tooth loss. [3] The development of a poly-microbial biofilm that forms as plaque on the tooth surface is the underlying cause of the disease. [4,5] In order to produce nutrition for their growth and function, periodontal pathogens produce degrading byproducts and enzymes that disintegrate host cell membranes and extracellular matrix. [6] Antimicrobial drugs both systemic and as in the form of local drugs has been administered and Antimicrobial medications have been shown to be quite successful in the treatment of bacterial illnesses. Bacterial pathogens, on the other hand, were quickly discovered to be resistant to many of the first effective medications. Therefore it is necessary to develop drugs that are effective against these putative pathogens. [7] Recently in the field of drug development the interest for herbal formulations and herbal medicine using different plant fragments and extracts has increased constantly. [8] The goal of developing herbal remedies was to reduce the resistance of some microorganisms to antimicrobial agents as well as to minimize unfavourable side effects and expensive treatment options. [9] Thousands of phytochemicals are produced by plants, and there are numerous methods to raise the quantities of bioactive products in the plant and get chemically assimilated extract. [10] *O. sanctum*, popularly referred to as tulsi in India, is said to be a significant foundation of the Ayurvedic holistic healing system. [11] The Indian subcontinent is home to the aromatic plant tulsi, which is a member of the Lamiaceae family. As a cultivar, tulsi is grown extensively. Because of its healing and spiritual qualities, tulsi is renowned for being called "The Queen of Herbs." Tulsi is grown for both medicinal and spiritual purposes in addition to being used to make essential oils. [12] Tulsi, also known as Ocimum sanctum (Linn.), has been a cornerstone of India's Ayurvedic holistic healthcare system. Several systemic disorders, including upper respiratory infections, bronchitis, skin conditions, malaria, etc., have long been treated using various plant parts. Antimicrobial Activity of *Ocimum sanctum L.* (Tulsi) has been evaluated against *Staphylococcus aureus,* *Proteus,* *Klebsiella,* *E. coli* and enteric pathogens. [13] Therefore, it is of interest to determine the antimicrobial efficacy of *Ocimum sanctum L.* (Tulsi) plant extract against anaerobic oral microbes.

## Materials and Methods:

**Figure 1:**
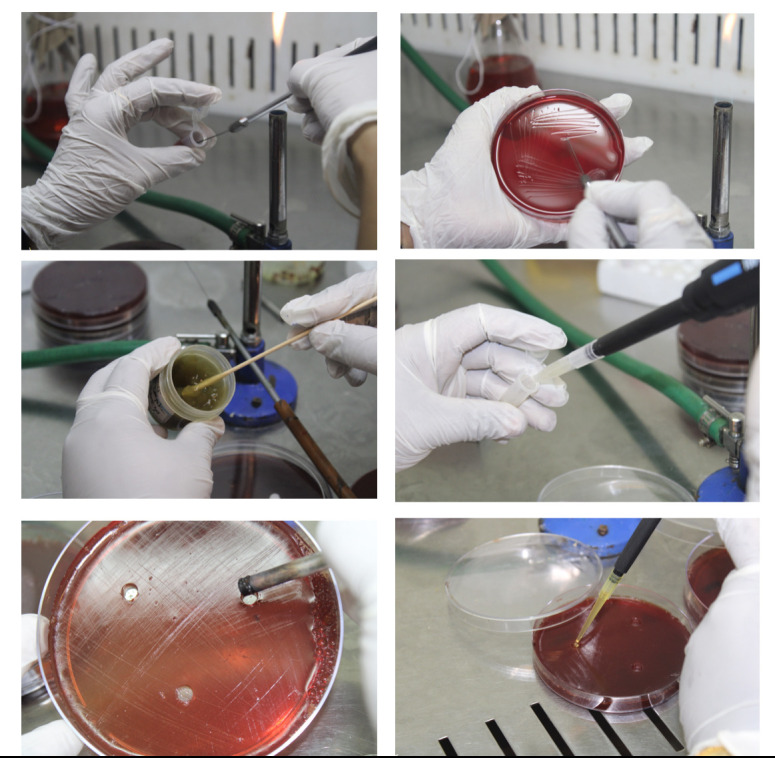
Agar well diffusion assay

Plaque samples were collected from the subject in sterile anaerobic blood broth and were reconstituted for 15 minutes. 10µL of sample was made as Lawn culture on to the sterile anaerobic blood agar plate using sterile swab (Figure 1). At 37° C the Plates were incubated for 5-7 days in the Gaspak anaerobic system. The colonies were counted after incubation. Number of colonies was recorded in the form of colony forming units per ml.

## Preparation of 2% *Ocimum sanctum L.* (Tulsi) gel

## Preparation of Supercritical fluid (SCF):

250 grams of *Ocimum sanctum L.* (Tulsi) powder is taken and soaked in 1000 mL of Ethyl alcohol for 48 hours. It is filtered with Whartman's filter. Filter liquid is evaporated that is Supercritical Fluid (SCF).

## Preparation of *Ocimum sanctum L.* gel:

Carbopol 940 was submerged overnight in distilled water that contained 0.2% sodium benzoate. HPMC solution, Propylene glycol and 2 ml of SCF (Homogenized) were added. Triethanolamine was added in drops and checked for pH. The pH ranges from 6-6.5. At room temperature, the *Ocimum sanctum L.* (Tulsi) gel was kept. For a period of six months, the *Ocimum sanctum L.* (Tulsi) gel that has been made is firm. Changes in pH were documented and corrected in accordance with the standard protocol.

## Antimicrobial activity of *Ocimum sanctum L.* (Tulsi) gel:

For the Antimicrobial efficacy of *Ocimum sanctum L.* (Tulsi) gel against the Total anaerobes was done by agar well diffusion assay. Briefly, Lawn cultures were made from the plaque samples using sterile swabs onto the sterile anaerobic blood agar plates. Wells were cut on the surface of the Agar. 50µL conc. of *Ocimum sanctum L.* (Tulsi) gel was added into each well. The Plates were incubated at 37° c by Gaspak system for 3-5 days. After incubation zone of inhibition was measured and recorded. The assay was repeated thrice and the mean value of the zone was taken as the inhibitory value.

## Results:

**Figure 2:**
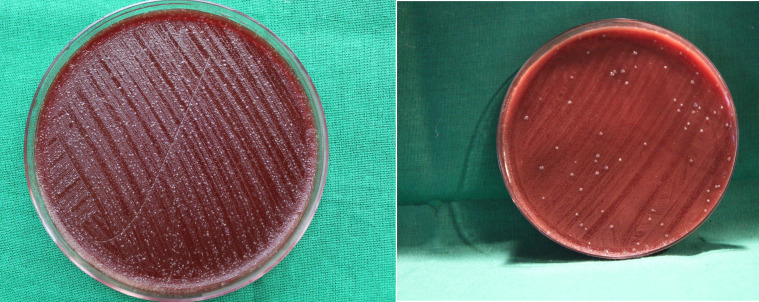
Anaerobic streptococci culture before placement of *Ocimum sanctum L.* (Tulsi) gel and after placement of *O. sanctum* gel (Left) before gel placement; (right) after gel placement

**Figure 3:**
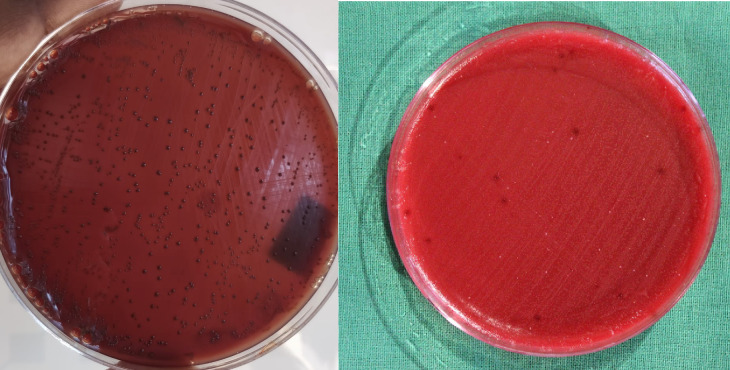
Black pigmented bacteroides culture Pre and post application of *Ocimum sanctum L.* (Tulsi) gel

**Figure 4:**
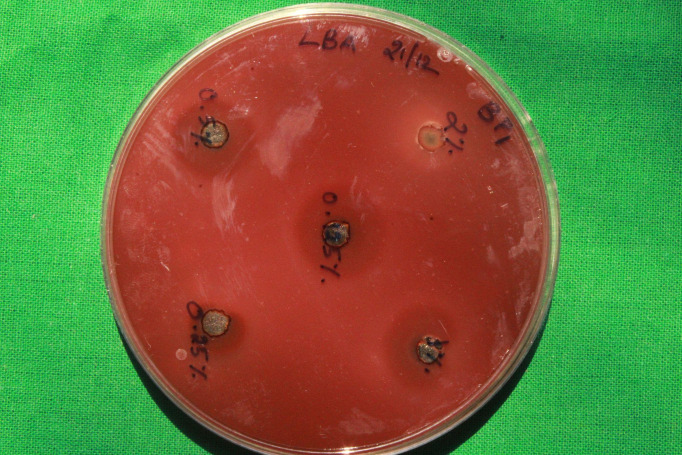
Post *Ocimum sanctum L.* (Tulsi) gel indicating the Zone of Inhibition in 5 different concentrations of *Ocimum sanctum L.* (Tulsi) gel

*Ocimum sanctum L.* (Tulsi) pretreatment and post treatment against anaerobic streptococci and black pigmented anaerobes (Table 2 and Figure 2, Figure 3, Figure 4). At the 1% w/v concentration of *Ocimum sanctum L.* (Tulsi) gel 25mm Zone of inhibition was obtained. At the 2%w/v concentration of *Ocimum sanctum L.* (Tulsi) gel 23mm Zone of Inhibition was obtained. At the 0.5%w/v concentration 20mm Zone of Inhibition was obtained. At the 0.25%w/v concentration of *Ocimum sanctum L.* (Tulsi) gel 15mm Zone of Inhibition was obtained. At the 0.125% w/v concentration of *Ocimum sanctum L.* (Tulsi) gel, an 18mm zone of inhibition was obtained (Table 3).

## Discussion:

Plant extracts have possible sources of new antimicrobial chemicals, particularly those that are effective against bacterial infections. The efficiency of the plant extracts in preventing bacterial growth varied, as evidenced by in vitro investigations. Numerous plant extracts' antimicrobial properties have previously been examined. They are divided into strong, medium, and weak categories. An important characteristic of plant extracts and their components is their hydrophobicity. [14] They make it possible for them to divide the membrane lipids of bacteria. They also make it possible for them to divide the mitochondria. Additionally, they disrupt cell structures and make them more permeable. The present study was conducted to assess the antimicrobial activity of Ocimum sanctum (Tulsi) extract against black pigmented anaerobes. *Ocimum sanctum L.* (Tulsi) demonstrated effective antimicrobial activity against Black pigmented anaerobes at 1%, 2% As higher the concentrations *Ocimum sanctum L.* (Tulsi) gel was effective, suggesting its possible use as an effective, and adjunct along with standard care in the management of periodontal condition. Our study results are in concordance with a study conducted by Gupta et al where he tested the effectiveness of a 4% w/v mouthrinse containing tulsi and 0.12% chlorhexidine in a triple-blinded, randomized controlled study. It was found that the mouthwash containing *Ocimum sanctum L.* (Tulsi) was equally efficient in reducing gingivitis similar to chlorhexidine, they have lower plaque levels. [15] According to Ipsita *et al.*, *Ocimum sanctum L.* (Tulsi) at an 8% concentration had the strongest antibacterial effects against A. actinomycetemco mitans and P. gingivalis. In order to control periodontal disorders, it is indicated that this can be effective as an addition to mechanical therapy. Ahirwar et al did a clinical study to determine a comparison between triple antibiotic paste and *Ocimum sanctum L.* (Tulsi) as a root canal treatment for primary molar teeth. Due to its antibacterial and anti-inflammatory properties, *Ocimum sanctum L.* (Tulsi) is recognized to produce superior results in long-term infections. It is therefore believed to be used as a root canal medication in primary dentition. [16] In vitro study conducted by Mallikarjun *et al.* stated that Periodontal microorganisms such Aggregatibacter actinomycetemco mitans, Prevotella intermedia, and Porphyromonas gingivalis are resistant to the antimicrobial effects of *Ocimum sanctum L.* (Tulsi) and doxycycline when used as a conventional treatment. 5% and 10% concentrations of *Ocimum sanctum L.* (Tulsi) demonstrated greater inhibitory zones against Aggregatibacter actinomycetemcomitans, he concluded. When tested against Porphyromonas gingivalis and Prevotella intermedia, they had narrower inhibitory zones. The use of *Ocimum sanctum L.* (Tulsi) as an effective adjunct and in addition to the usual periodontal treatment is thus demonstrated. [17]

Parida *et al.* [18] Using the Soxhlet apparatus, ethanol extract was produced from *Ocimum sanctum L.* (Tulsi) leaves. Using the broth dilution method and agar well diffusion, the concentration of 400 µg/ml was chosen to assess the antimicrobial effects against common oral microbes such as *Lactobacillus acidophilus,* *Streptococcus mitis,* *Candida albicans,* *Prevotella intermedia,* *Streptococcus mutans,* and *Pepto streptococcus.* Streptococcus and *Streptococcus* were both inhibited by the ethanolic extract at a concentration of 400µg/ml with an inhibitory zone of 7.33 mm for each. *Pepto streptococcus* and *P. intermedia* resistance to the extract is seen. Moreover, it was effective against candida (zone of inhibition was 10.67 mm). On Lactobacillus, there was, however, no inhibitory impact. Oral microbes were discovered to be inhibited by *Ocimum sanctum L.* (Tulsi) extract. Ramamurthy *et al* conducted a study about Gel made from *Ocimum sanctum L.* (Tulsi) that may have anti-inflammatory and antioxidant qualities. It has a lower toxin level than brine shrimp nauplii. The most effective agent for treating periodontitis was *Ocimum sanctum L.* (Tulsi). [19] Study done by Deepika *et al.*, [20] demonstrated that *Ocimum sanctum L.* (Tulsi) demonstrated effective antimicrobial activity against anaerobic oral microbes at 20%, 25%. As higher the concentrations Tulasi gel is effective, suggesting its possible use as an effective, and adjunct along with standard care in the management of periodontal condition. Chlorhexidine gel showed no growth when compared to Tulasi gel. Deepika *et al.*, [21] 2% of *Ocimum sanctum L.* (Tulsi) showed that it is effective in reducing gingival bleeding and gingival inflammation. It also helps in reducing the Plaque. *Ocimum sanctum L.* (Tulsi) showed no side effects when compared to Chlorhexidine (CHX).

Periodontal disease and tooth loss have been linked to black-pigmented Gram-negative anaerobes. Deep periodontal pockets are closely associated with its presence and are thought to be its primary habitat. Moreover, correlations with clinical inflammation, attachment loss, and serum antibody levels have been found, suggesting an aetiological significance in the periodontal disease. [22]

## Conclusion:

*Ocimum sanctum L.* (Tulsi) demonstrated effective antimicrobial activity against Black Pigmented microbes at 1% and 2% concentration. Growth of anaerobic streptococci and black pigmented anaerobes reduced significantly after the application of *Ocimum sanctum L.* (Tulsi) gel. Nature based products can be preferred for the treatment of periodontal disease as they have lesser side effects. *Ocimum sanctum L.* (Tulsi) gel can be used as an adjunct to standard care in the management of periodontal disease. However, long term clinical trials are required to validate the results.

## Figures and Tables

**Table 1:** The ingredients of preparation of 2% *Ocimum sanctum L.* (Tulsi) gel

**INGREDIENTS**	**QUANTITY**
Carbopol 940	2g
Polymer (HPMC)	2g
Tulsi SCF extract	2ml
Sodium benzoate	0.2ml
Propylene glycol	5ml
Triethanolamine	q.s
Distilled water	q.s to make 100ml
HPMC- Hydroxy Propyl Methylcellulose;
SCF - Super Critical Fluid

**Table 2:** *Ocimum sanctum L.* (Tulsi) pre-treatment and post treatment against anaerobic streptococci and black pigmented anaerobes

**Bacteria**	**Pre *Ocimum sanctum L.* (Tulsi) gel**	**Post *Ocimum sanctum L.* (Tulsi) gel**
Anaerobic Streptococci	1*104	6.8*102
Black Pigmented Bacteroides	2.7*102	1.5*101

**Table 3:** Zone of Inhibition of Black Pigmented Anaerobes with *Ocimum sanctum L.* (Tulsi) gel at 5 different concentrations

**Concentration of *Ocimum sanctum L.* (Tulsi) gel**	**Zone of Inhibition**
1%	25mm
2%	23mm
0.50%	20mm
0.25%	15mm
0.13%	18mm
